# Exploring the pattern of use and accessibility of urban green spaces: evidence from a coastal desert megacity in Saudi Arabia

**DOI:** 10.1007/s11356-022-19639-4

**Published:** 2022-03-23

**Authors:** Abdullah Addas

**Affiliations:** Landscape Architecture Department, Faculty of Architecture and Planning, King Abdulaziz University, P.O. Box 80210, Jedd+10ah, 21589 Saudi Arabia

**Keywords:** Urban green spaces, Ecosystem services, Urban parks, Jeddah megacity, Social relation, Climate change

## Abstract

Urban green spaces (UGSs) provide various ecosystem services (ESs) that directly and indirectly enhance people’s well-being. However, in the Saudi context, the assessment role of UGSs (such as urban parks and gardens) and their use and accessibility has remained unexplored. This study aims to assess the use and accessibility of five urban parks in the Jeddah megacity of Saudi Arabia from diversified perspectives. Data were collected through a primary survey and questionnaire method using a social preference approach (SPA). Correlation analysis and factor analysis were performed to assess the relationship between activities and services (benefits) provided by urban parks and to examine their most significant benefits. A Kruskal–Wallis (K–S test) test was performed to determine significant differences in the perceived valuations of park benefits. A benefit dominancy index (BDI) was also developed to determine which urban parks provide the most benefit. The findings of the study showed that (i) the urban parks were mostly used for spending time with relatives (partners) and friends, followed by mental refreshment and relaxation, physical activity, and spending time with children; (ii) there are substantial seasonal variations in park visits in the Jeddah megacity; (iii) socio-demographic attributes largely affect the use of urban parks; and (iv) there are also substantial discrepancies between importance and performance related to urban management strategies. Thus, the findings of this study show that city planners and policy makers must focus on the enhancement of UGSs for the well-being of urban citizens.

## Introduction

As per the estimation of the United Nations (2018), the population of urban area residents is likely to reach 68% by 2050, resulting in a loss of natural habitat for other organisms, as rapid urban expansion has become one of the major drivers of the loss of green spaces (Wu et al. 2019; Nor et al. 2017; Xu et al. 2019). However, urban green spaces (UGSs) play a crucial role in local climate regulation (Finaeva 2017), enhance the quality of soil (Setala et al. 2017), support physical and metal benefits (Konijnendijk et al. 2013; Sturm and Cohen 2014), and improve social cohesion, social inclusion, and interactions (Peters et al. 2010; Kazmierczak and James 2007). Recently, a number of studies have been performed in cities across the world, such as Singapore (Henderson 2013), Tokyo (Kohsaka and Okumura 2014), New York (Sutton and Anderson 2016), and Delhi (Paul and Nagendra 2017), and these studies showed that green spaces (GSs) play a crucial role in regulating urban health (Huang et al. 2017; Lee and Maheswaran 2011; Zhang et al. 2017), quality of life (McFarland et al. 2008; Artmann et al. 2017; Sanesi and Chiarello 2006), and maintaining the bio-diversity and sustenance of livelihoods (Gunnarsson et al. 2017; Raymond et al. 2018; Devisscher et al. 2019).

ESs are the direct and indirect benefits that humans obtain from ecosystems (MEA 2005). UGSs, within the urban environment, provide various direct and indirect ecosystem services (ESs) to the urban population, such as stress relief and health improvements (D’Souza and Nagendra 2011; Niemelä et al. 2010; Cilliers et al. 2013; Ko and Son 2018; Enssle and Kabisch 2020). Thus, the quality of life of urban citizens can be improved through the enhancement of GSs within a city (Mensah et al. 2016). In previous studies, it has been extensively documented that GSs within the urban environment play a crucial role in the well-being of urban citizens (Ma et al. 2019; Kothencz et al. 2017; Carrus et al. 2015). Within urban ecosystems, urban parks are considered significant ecosystems, as they provide various services, such as air purification, water purification, the reduction of noise and wind, the regulation of micro-climate, habitat for wildlife, and social and psychological well-being of urban citizens (Jennings et al. 2016; Sun et al. 2019; Misiune et al. 2021). Urban citizens benefit from urban parks, and the presence of GSs enhances their quality of life (Kabisch and Haase 2014), e.g., through the alleviation of mental stress (Braubach et al. 2017; Tsai et al. 2018) and the improvement of mental well-being (Wang et al. 2019; Du et al. 2021). However, the ecological values of GSs are limited due to their size and artificiality (Cilliers et al. 2013).

Interactions between humans and urban parks have been widely studied from the perspectives of various groups of park users, focusing on demographic attributes, social statuses, and ethnic groups (Gobster 2002; Elmendorf et al. 2005; Breuste and Rahimi 2015; Arnberger et al. 2017; Artmann et al. 2017). Recently, studies have been performed on urban park preferences, largely implemented into an ecosystem service framework (Bertram and Rehdanz 2015; Buchel and Frantzeskaki 2015). The accessibility and use of urban parks is largely influenced by the attitudes of visitors towards urban GSs (Balram and Dragićević 2005). Visits to urban parks are largely determined by their physical features, vegetation cover (Shanahan et al. 2015), psychological factors (Wan et al. 2020; Gomez et al. 2015), and diverse facilities (Wan et al. 2020). The perceived benefits from urban parks are influenced by the actual benefits obtained from them and their perceived importance (Wan et al. 2020). Thus, in this context, effective management strategies can be adopted based on perceived importance, accessibility, and benefits.

The basic research question that arises in this context is as follows: What factors affect the perceived importance and benefits of urban parks? In previous studies, attempts have been made to examine the relationship between park visitors and GS environments (Gozalo et al. 2019; Knobel et al. 2021; Pietilä et al. 2015; Akpinar, 2016; La Rosa et al. 2018). Attitudes towards parks, their use, and the physical activities conducted therein are determined by their physical features, such as trees, sport facilities, water facilities, and playgrounds (Sang et al. 2016; Wan and Shen 2015; Baran et al. 2018; Kim and Jin 2018). A relatively high quality of GSs and perceived safety contribute positively to the use of GSs and are crucial for meeting social and psychological demands (Sanesi and Chiarello 2006; Giles-Corti et al. 2005; Haq 2011). According to van Dillen et al. (2012), GSs with high psychological, physical, and social benefits were considered pleasurable, attractive, and safe. Thus, it can be stated that the relationship between visitors and GSs is influenced by two prominent factors, namely, the physical attributes of the parks (such as vegetation cover and infrastructural facilities) and the psychological attributes of the visitors (such as perception towards safety and aesthetic values) (Wan et al. 2020). Therefore, it is urgent to focus on the perceived valuation and benefits of urban parks as well as their accessibility and use to improve quality of life and the management of GSs within urban environments.

In the last few decades, Saudi cities experienced rapid urban expansion that has resulted in substantial land use and land cover (LULC) change (Alqurashi and Kumar 2016, 2019). This transformation of LULC has affected the thermal environment of cities and caused the emergence of urban heat islands (UHIs) (Niu et al. 2020; Detommaso et al. 2021). At the same time, climate change has become a serious challenge to city planners and policy makers. The effective management and restoration of green spaces in urban environments require crucial, nature-based solutions. Apart from this, green spaces, particularly urban parks and urban gardens, play a significant role in the enhancement of the quality of life of urban citizens. In the study reported in this paper, an attempt was made to examine the socio-ecological relationship between humans and urban parks in a desert megacity. Despite the significant impact of GSs on the well-being of urban citizens, Saudi cities are facing great challenges in encouraging people to appreciate the benefits obtained from GSs. Jeddah is one of the largest megacities in Saudi Arabia, and the per capita availability of GSs is much lower than in other megacities of Saudi Arabia. Addas and Maghrabi 2021a) reported that the per capita availability of GSs was 0.5 m^2^ in Jeddah, which is much lower than that of other Saudi megacities, such as Riyadh and Dammam. Therefore, it is essential to study the use and accessibility of urban parks and the socio-ecological relationship between humans and urban parks, and to implement effective accessibility policies to improve the well-being of urban citizens in order to (i) meet the needs of urban citizens and enhance their quality of life and (ii) cope with climate change through nature-based solutions. To the best of our knowledge, no studies have been performed on the use or accessibility of urban parks through social perception and preferences in the context of Saudi cities. Considering this research gap, this study aims to examine the patterns of the use and accessibility of urban parks in the Jeddah megacity. The findings of this study will assist city planners and policy makers in understanding and implementing effective measures to manage and restore green spaces, particularly urban parks and gardens.

## Material and methods

### Study area

**Fig. 1:**
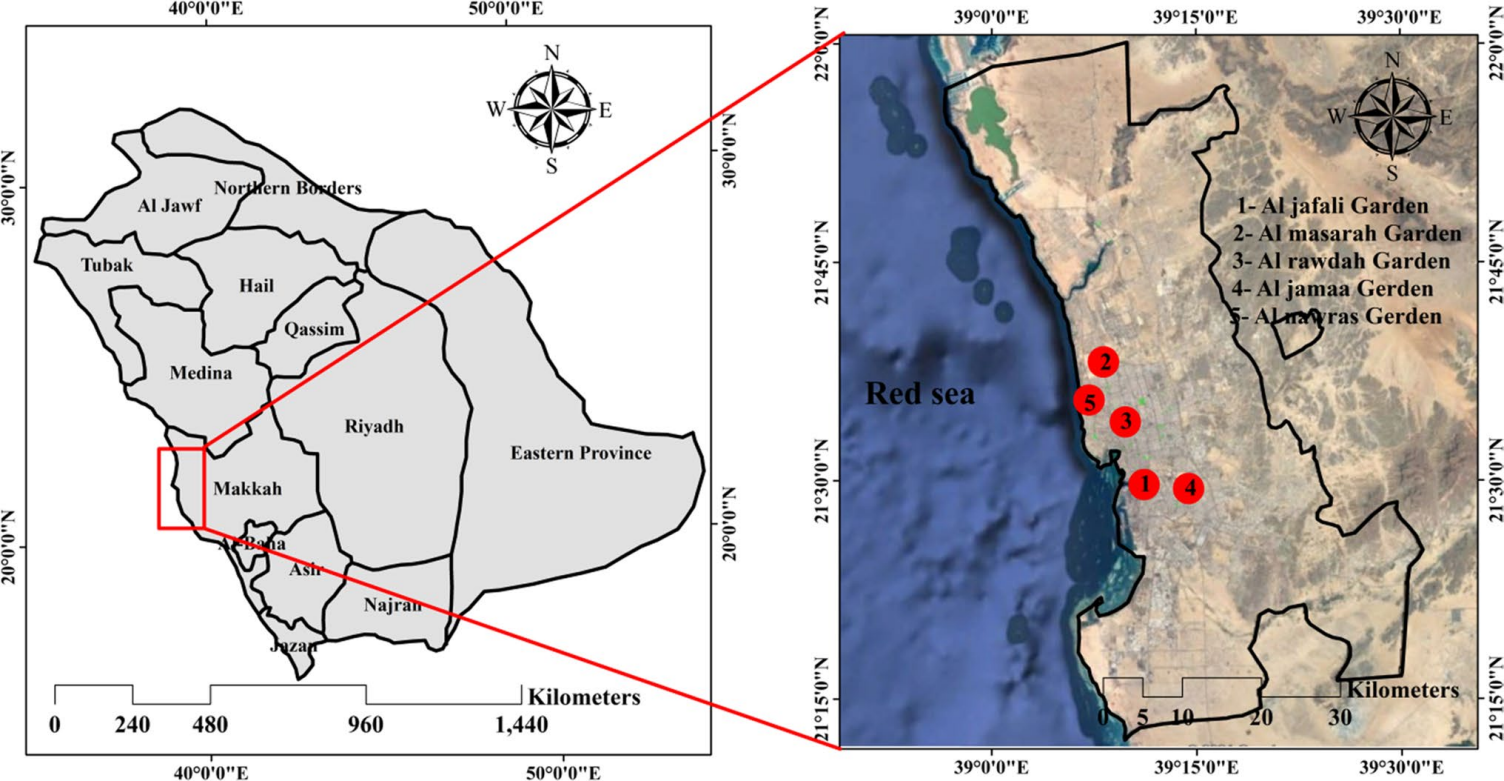
Locations of urban parks in the Jeddah megacity

Jeddah, located on the eastern coast of the Red Sea, is one of the largest megacities in Saudi Arabia. The total population of Jeddah is 4.276 million with a population density of 2670/km^2^. Geographically, Jeddah is located in the eastern part of the Red Sea and has a dry and hot desert climate. The total area is about 1600 km^2^. The average temperature is around 28 °C during winter and over 40 °C during summer. The average annual rainfall is about 45 mm. In recent decades, the Jeddah megacity has experienced rapid urban expansion and is one of the most rapidly growing cities in Saudi Arabia. Figure 1 shows the locations of urban parks in Jeddah.

### Questionnaire survey and data collection

Park visitors were selected as per a pre-tested questionnaire. In five urban parks, 409 visitors were surveyed face to face in 2021 using a simple random sample. The parks were selected from different parts of the city for a better understanding of the spatial social variation in urban parks. The roles and importance of GSs are crucial during the hot summer season. Therefore, the survey was performed during the summer season from March to May in 2021. A questionnaire survey was administered on public holidays and weekends. A pilot survey was also performed to validate the questionnaire selected for the study and to avoid ambiguity. After the pilot survey, the irrelevant questions were removed from the questionnaire and questions were revised accordingly. The survey was also conducted within specific time periods: from 6 a.m. to 9 a.m. during the morning and from 4 p.m. to 8 p.m. during the evening, due to the higher concentration of park visitors at these times. The questions were initially developed in English, but were translated into Arabic, as most of the park visitors tend to speak Arabic. Approximately, 10% of the total park visitors were non-Saudi, and questions were asked to them in English. Each interaction lasted approximately 15 to 20 min. The semi-structured questionnaire for the collection of data had four major sections: (a) Section I: general information about the respondents (such as gender, age, educational level, and occupation); (b) Section II: major activities carried out within the urban parks; (c) Section III: benefits obtained from the urban parks; and (d) Section IV: management strategies related to urban parks.

For the selection of visitors, the following equation was used (Dillman 2000):

1 $$\mathrm{n}={\left[\left(\mathrm{N}\right)\left(\mathrm{p}\right)\left(1-\mathrm{p}\right)\right]/[(\mathrm{N}-1)(\mathrm{B}/\mathrm{C})}^{2}+(\mathrm{p})(1\mathrm{p})]$$

where n is the sample size for the study, N is the total expected visitors for each urban park, p is the proportion of park visitors, B is the sampling error, and C indicates the Z statistic associated with the confidence level, which is 1.96, corresponding to the 95% level. The total number of park visitors selected was 409 and details are presented in Table 1.

### Identification of activities (or services) obtained from parks and management attributes

In this study, 12 activities or services provided by the urban parks were identified. The activities or services carried out in urban parks were identified based on the previous literature (Basu and Nagendra 2021; Ko and Son 2018) and direct field observation. These details of the activities or services are presented in Table 2.

**Table 1:** Detailed overview of the selected urban parks

Name of park	Expected visitors	Approx. area (m^2^)	Type of park (public/private)	Foundation year	Number of yampled respondents
Al JafaliGarden	More than 100	8500	Public	2000	95
Almasarah Garden	More than 101	44,250	Public	2008	104
Alrawdah Garden	More than 100	16,524	Public	2002	86
AljamaaGerden	More than 200	55,380	Public	2017	111
AlnawrasGerden	More than 100	20,000	Public	2018	73

**Table 2:** Details of activities (or services) carried out in urban parks

Activities (or services)	ID	Description
Walking	A	Urban parks provide opportunities for walking, e.g., walking for physical fitness and walking with friends and relatives
Natural environment	B	Urban parks as green spaces provide sufficient opportunities to enjoy the environment
Experience nature and its aesthetic beauty	C	In cities, there are limited opportunities to experience nature, particularly in densely populated cities. Thus, urban parks provide opportunities to experience nature and its aesthetic beauty
Spend time with relatives (partner) and friends	D	Outdoor spaces are very important in cities to spend time with friends and relatives
Mental refreshment and relaxation	E	Urban parks as urban green spaces enhance mental health through increased opportunities for mental refreshment and relaxation
Spend time with children (playing, traveling)	F	Urban parks are vital for the socialization of children and provide opportunities for children to spend time with other children and adults (e.g., family, neighbors)
Avoid loneliness	G	Urban parks as green spaces provide opportunities to avoid loneliness, particularly for the elderly, and to improve mental health
Reading	H	Urban parks provide opportunities for reading and learning in a natural environment
Picnic	I	Urban parks are widely used for picnicking with friends and families on the weekend
Physical activity	J	Urban parks are important for physical health. They are used for jogging, walking, running, and other physical fitness activities
Sports	K	Urban parks are used for sports and provide opportunities for social bonding for adults and children
Social relations	L	Urban parks are very important for strong social cohesion with friends and neighbors and help in building social bonds

In this study, park visitors’ perceptions of management strategies were assessed to understand the satisfaction of park visitors with park facilities. The perception of management strategies was assessed based on a 5-point Likert scale ranging from 1 to 5, where 1 indicates very low importance or strongly disagree, and 5 indicates very high importance or strongly agree. To evaluate the perception of management strategies, visitors were asked to rate its importance (ranging from 1 to 5) and provide a performance score (ranging from 1 to 5). Twelve attributes related to management strategies of the urban parks were assessed. This assessment is valuable in facilitating an understanding of the discrepancies between the importance and performance of management strategies, based on which policies can be promoted.

### Development of benefit dominancy index (BDI) of the urban parks

In this study, the benefit dominancy index (BDI) was developed to semi-quantitatively examine the most significant benefits obtained from the five urban parks. For the assessment of the BDI, a score was assigned as significantly positive (+ +), positive ( +), neutral (0), negative ( −), significantly negative (− −), or unknown (?) (Table 3). For the development of the BDI, the total number of benefits provided by the urban parks (*n* = 12) was summed up and further divided into benefits falling in certain groups (such as significantly positive or positive). The following equation was used to develop the BDI:

$$BDI=\frac{\updelta \left({\mathrm{n}}_{+1.0}+{\mathrm{n}}_{+0.5}\right)+ \delta ({n}_{-1.0}+ {n}_{-0.5})}{{\delta benefits}_{total}}$$

where *n*_+*1.0*_ is the number of benefits assigned as significantly positive, *n*_+*0.5*_ is the number of benefits assigned as positive, *n*_*-1.0*_ is the number of benefits assigned as significantly negative, *n*_*-0.5*_ is the number of benefits assigned as negative, and *benefits*_*total*_ is the total number of benefits (*n* = 12) used in this study. The value of BDI ranges from + 1 to − 1, where a value close to + 1 indicates a higher dominancy of benefits and a value close to − 1 indicates a lower dominancy on benefits. The visitors were asked to provide their responses based on the benefits obtained from the parks. The benefits obtained from the parks were categorized into five classes ranging from significantly positive to significantly negative.

**Table 3:** Scaling for the development of the BDI

Assigned importance	Significantly positive	Positive	Neutral	Negative	Significantly negative	Unknown
Importance score	+ +	+	0	−	− −	?
Numerical value	1	0.5	0	− 0.5	− 1	Removed from analysis

### Statistical analysis

In this study, several statistical analyses were performed for the multi-scale assessment of urban parks. The Kolmogorov–Smirnov (K-S) test was used for the assessment of the normality of the data (*p* > 0.05). We used a non-parametric test, the Kruskal–Wallis test (K-S test), to determine the difference in the perception and satisfaction of park visitors due to a lack of normality (*p* > 0.05). The correlation coefficient determined relationships among benefits provided by urban parks. Factor analysis was also performed to examine the prominent benefits. All statistical analyses were performed using the SPSS software (version 22).

The perceived valuations were also assessed based on a 5-point Likert scale. The perceived importance of the benefits was assessed as very high (assigned as 5), high (assigned 4), moderate (assigned as 3), low (assigned as 2), or very low (assigned as 1). The perceived importance of the benefits obtained from urban parks was assessed to examine the impact of socio-demographic attributes on the benefits of urban parks.

## Results

### Profile of the park visitors

This study included visitors with diverse socio-demographic attributes (such as gender, age, occupation, and educational level) (Table 4). The sample was 56% of males and 44% of females across five parks, with the highest proportions of male and female visitors being from Al Masarah Garden (61% male) and Al Rawdah Garden (49%), respectively. The dominant age group surveyed was between 30 and 40 (32% of the total park visitors), followed by the age groups between 20 and 30 (28%), 40 and 50 (22%), and above 50 (17%). The highest percentage of the age group between 30 and 40 was reported in Al Jamaa Garden (38%), followed by Al Jafali Garden (34%) and Al Masarah Garden (33%). Park visitors with a high educational status (mostly a bachelor’s degree or higher) figured prominently among park visitors in Jeddah. For example, among all the respondents from the parks, 41% of visitors were educated with a bachelor’s degree or higher (29%), a high school education (19%), or an elementary school education (9%). In the case of occupations, most of the park visitors were government employees (30%), followed by businessmen (28%), homemakers (19%), and students (18%). Most visitors came to the parks with their partners (30.6%), but some came with friends (20.4%), family members (17.6%), or children (15.8%). A total of 39% of visitors came to Al Nawras Garden with a partner or spouse, whereas only 23% of visitors came to Al Rawdah Garden on their own.

**Table 4:** Socio-demographic profile of visitors in five urban parks (%)

Dimension	Socio-demographic attributes	Urban parks				
		Al Jafali garden (*N* = 95)	Al Masarah garden (*N* = 104)	Al Rawdah garden (*N* = 86)	Al Jamma garden (*N* = 111)	Al Nawras garden (*N* = 73)
Gender	Male	56	61	51	55	57
	Female	44	39	49	45	43
Nationality	Saudi	94	96	96	97	99
	Non-Saudi	6	4	4	3	1
	20–30	26	29	34	24	31
	30–40	34	33	26	38	28
	40–50	22	23	19	22	24
	> 50	18	15	21	16	17
Educational level	Elementary	12	10	8	9	10
	High school	19	21	21	20	16
	Bachelor	38	46	37	41	46
	Above masters	31	23	34	30	28
Occupation	Students	22	16	22	14	18
	Government employee	26	32	37	33	27
	Businessman	33	22	19	34	42
	Homemaker	19	30	22	19	13

Source: Field survey, March to May 2021

### Pattern of urban use or activities carried out in parks

**Fig. 2:**
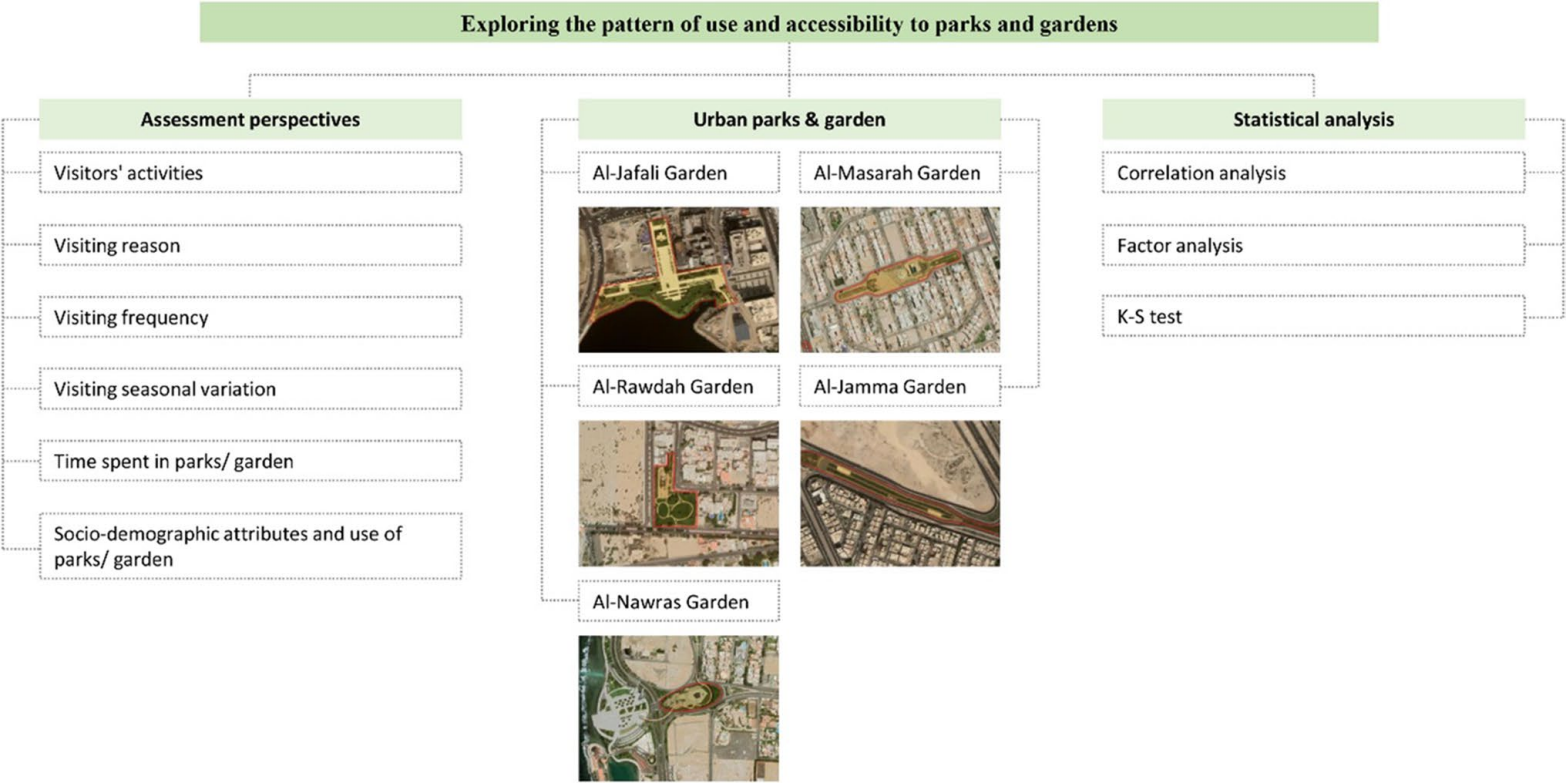
Methodological framework of the study

**Fig. 3:**
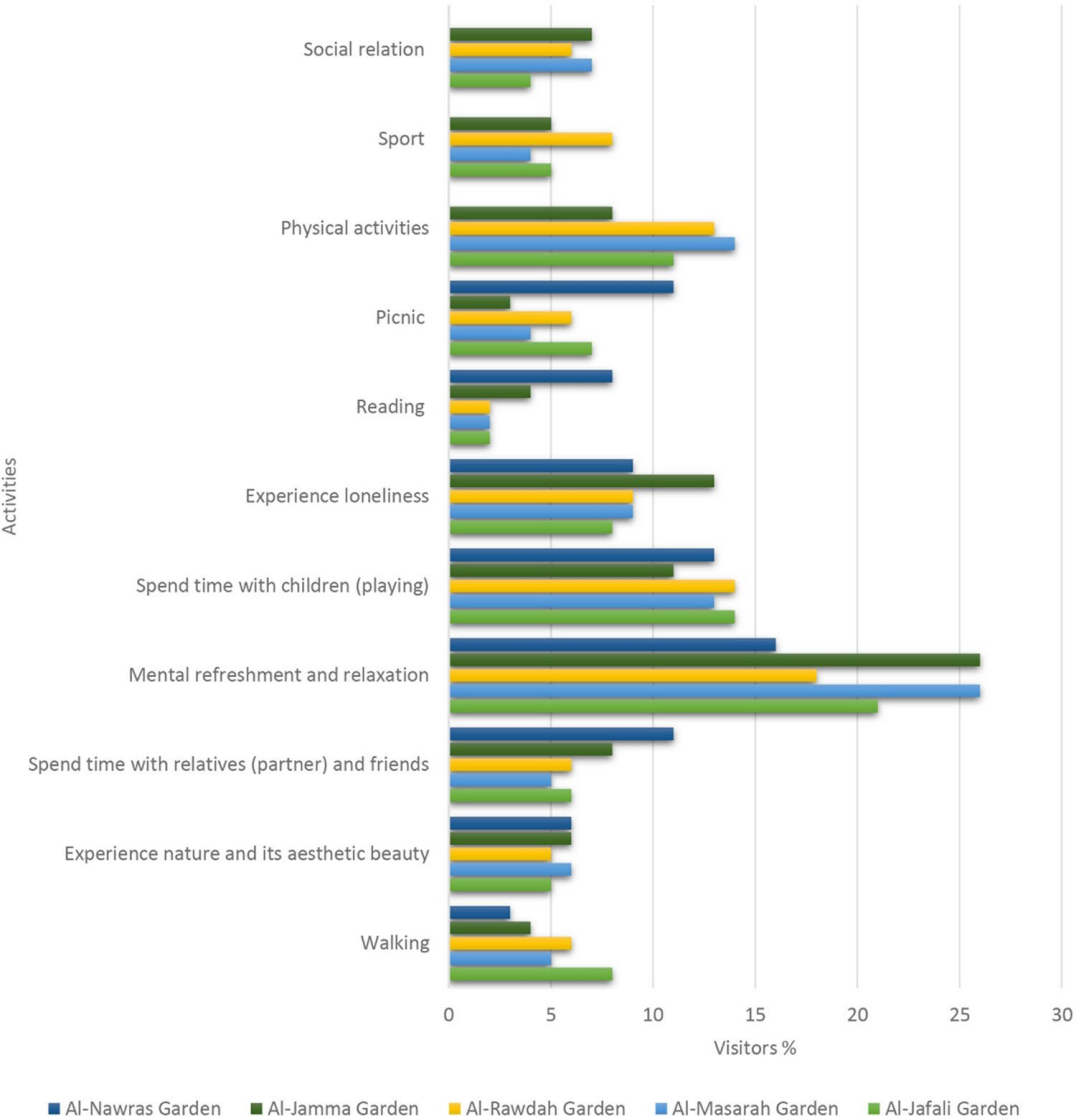
Reasons for visiting parks in Jeddah

**Fig. 4:**
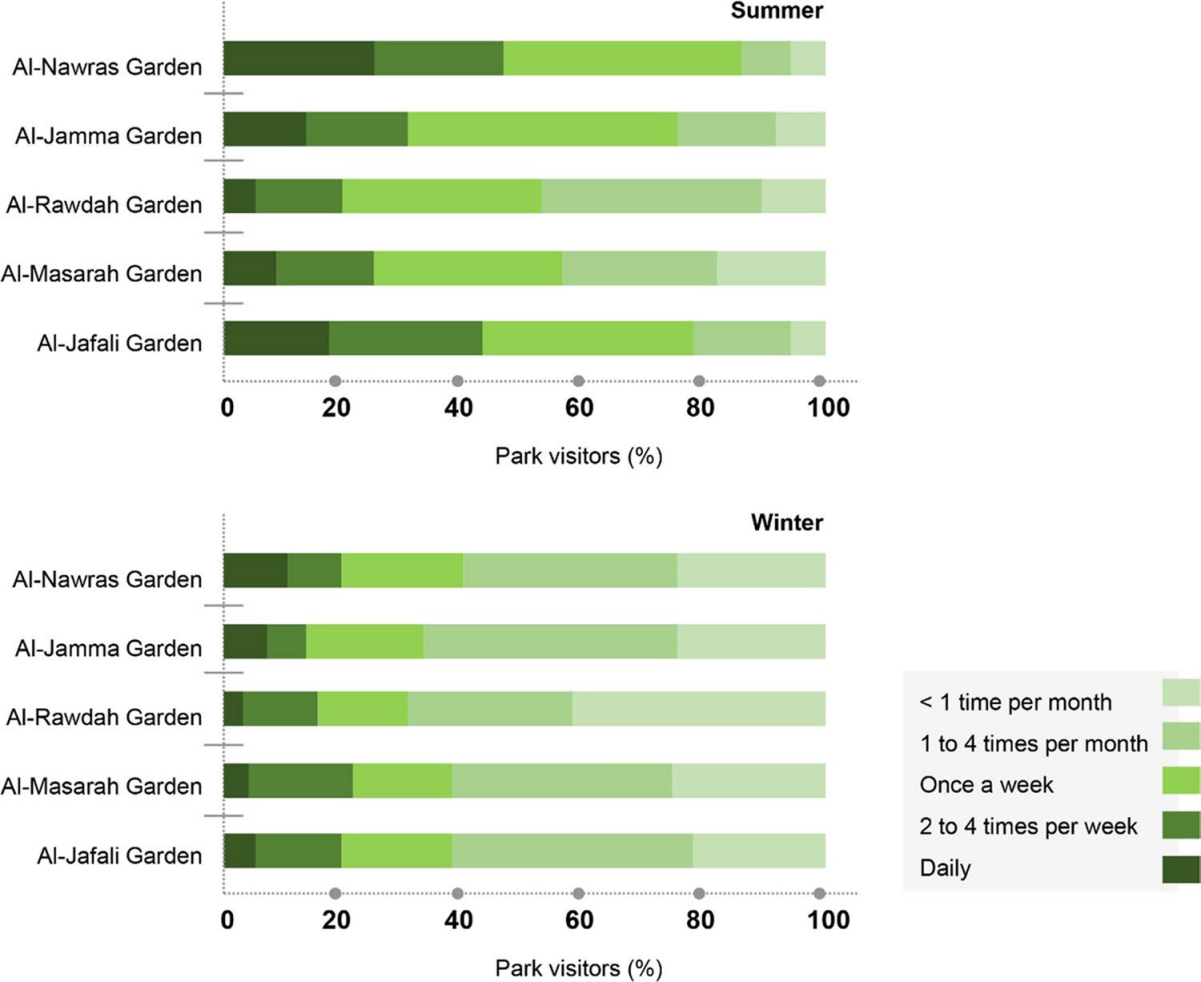
Seasonal variation in park visitors (%) in five gardens/parks

**Fig. 5:**
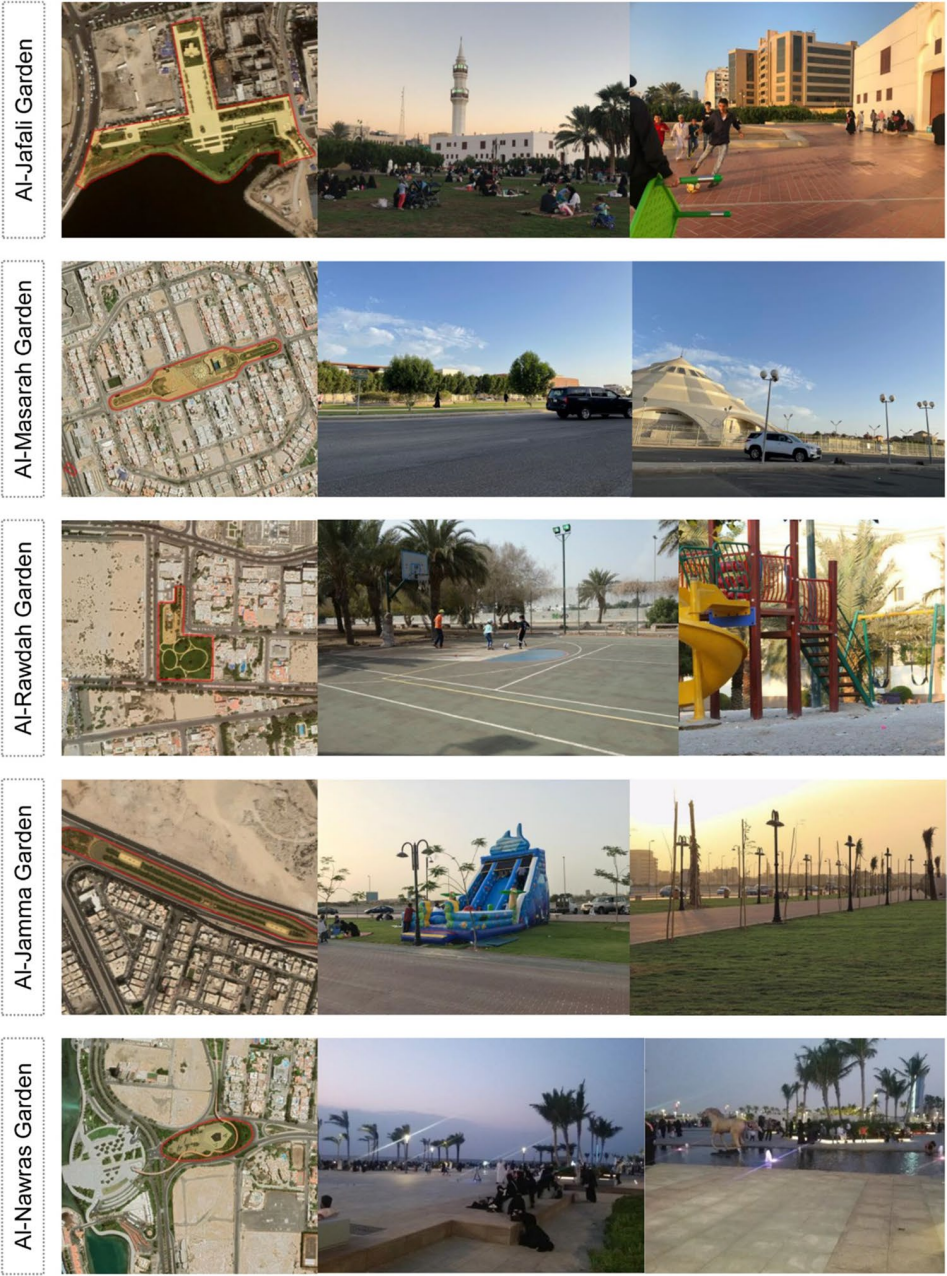
Field photos from five urban parks: Al Jafali Garden (Al Masarah Garde, Al Rawdah Garden, Al Jamaa Garden, and Al Nawras Garden)

It was found that urban parks provided diversified benefits to the visitors. Based on an overall analysis, urban parks were mostly used for spending time with relatives (e.g., partners) and friends (21.26%), followed by mental rejuvenation and relaxation (13%), physical activity (11.82%), spending time with children (9.58%), experiencing nature and its aesthetic beauty (7.36%), and picnics (6.06%). There were slight differences in terms of activities in each separate park. For example, the highest percentage of park visitors spending time with relatives (partners) and friends was reported in Al Masarah Garden (26%), followed by Al Jamaa Garden (25.3%), and Al Jafali Garden (21%). The highest percentage of park visitors engaging in physical activity was reported in Al Nawras Garden (14.6%), followed by Al Jafali Garden (13.3%), Al Rawdah Garden (11.9%), Al Masarah Garden (11%), and Al Jamaa Garden (8.3%). The results showed that urban parks in Jeddah are rarely used for reading (2.96%), sports (5.12%), and social relations (5.22%) (Figs. 2, 3, 4 and 5).

### Pattern and importance of urban park use

#### Seasonal variation and duration of park visits

**Fig. 6:**
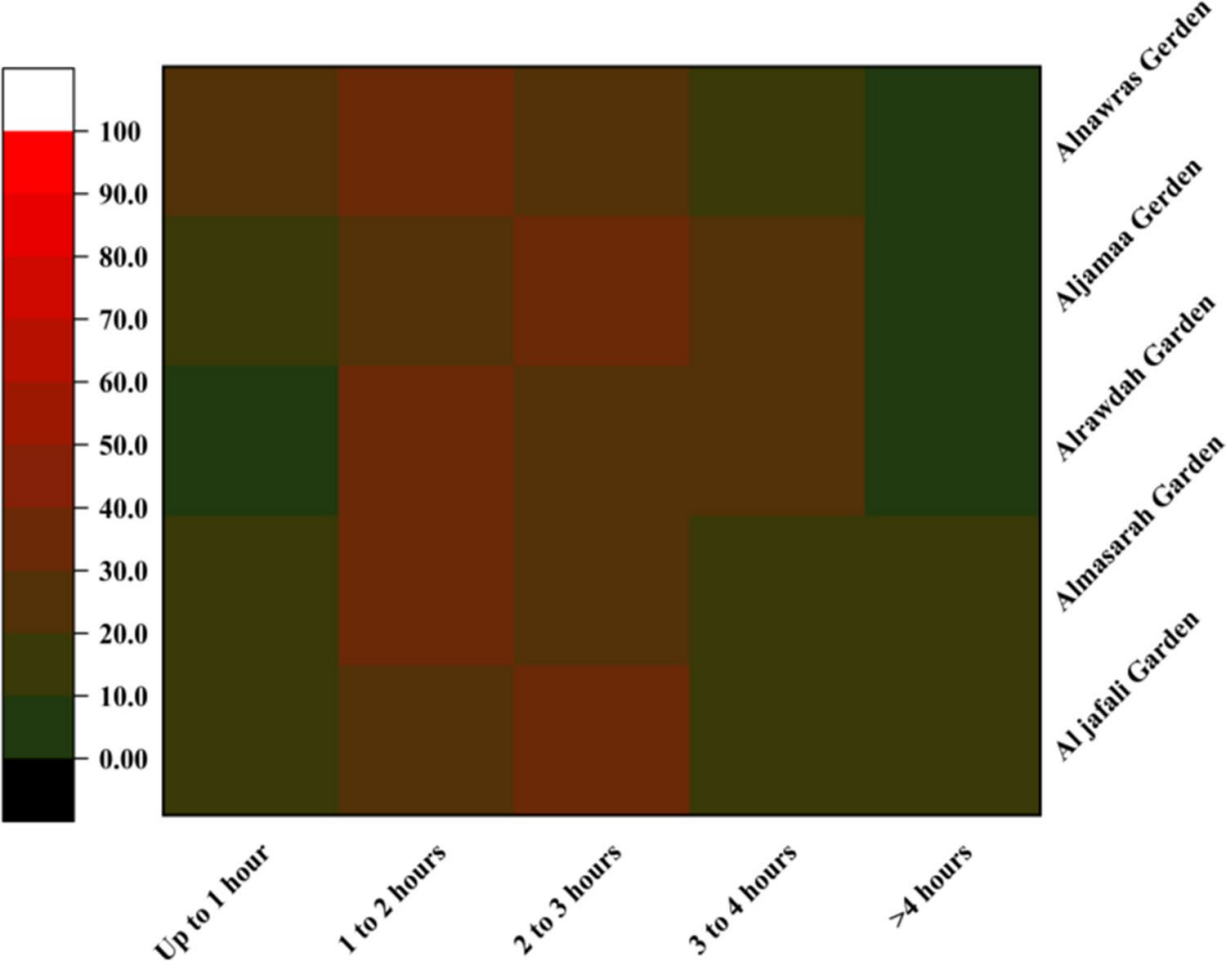
Time spent in parks (%) during the summer

**Fig. 7:**
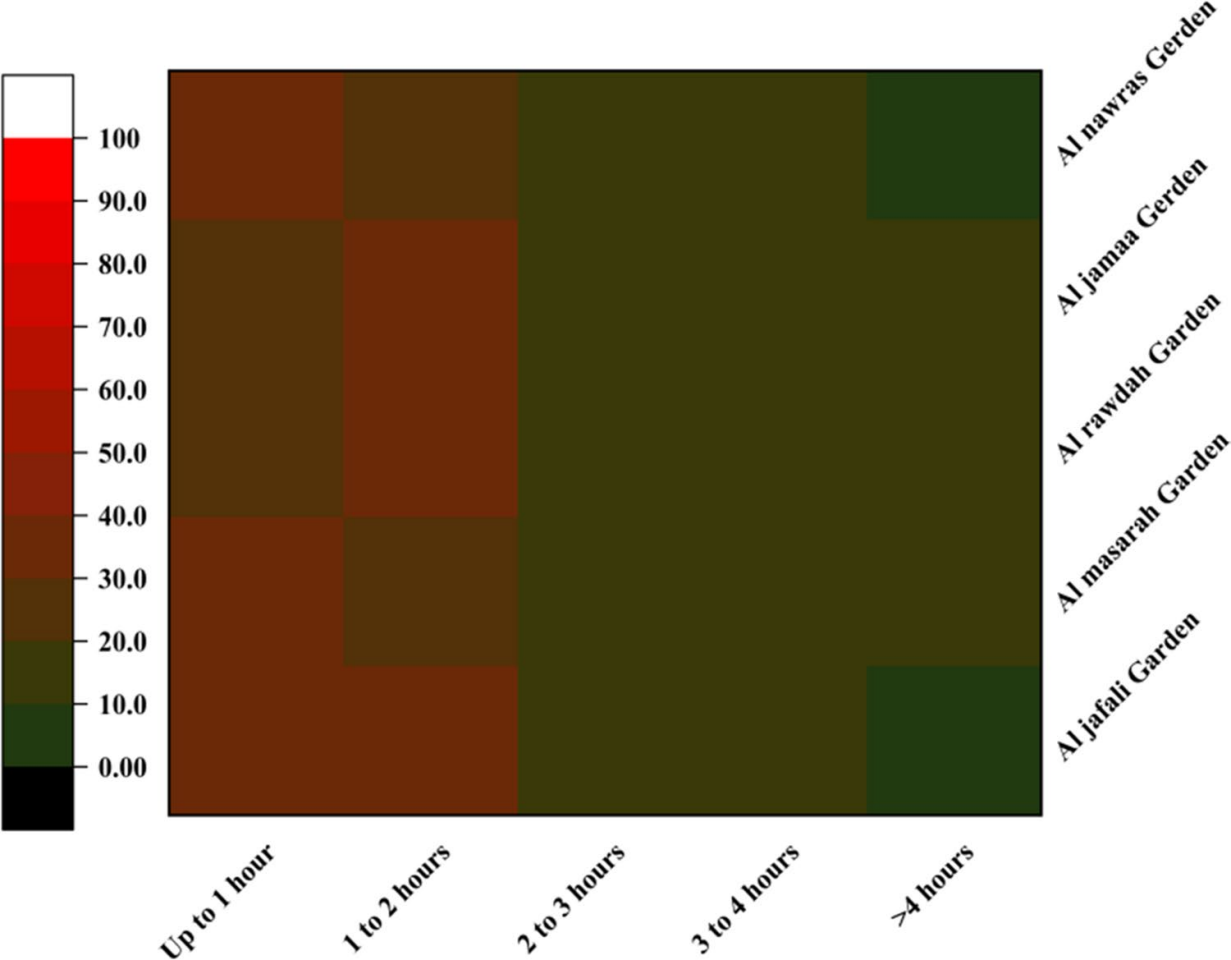
Time spent in parks (%) during the winter

In this study, an attempt was made to examine the frequency and duration of urban park use. This study was performed during the summer season, but visitors were also asked about the time spent in the parks during the winter season. The results show that there is a substantial difference in the frequency and duration of urban park use across the urban parks, along with seasonal variation. The frequency and duration of park use were higher in the summer. About 30% of the total visitors spent 1–3 h in the parks in the summer but only 22% of the total visitors reported that duration in the winter (Figs. 6 and 7). More than 32% of the total visitors spent less than 1 h in parks during the winter, whereas only 14.4% of the total visitors reported that duration in the summer. Substantial differences were also reported in the frequency of park visits. For example, the percentages of daily (15.2% in summer and 8% in winter) and weekly (34.8% in summer and 17.4% in winter) visitors were higher in the summer (Fig. 4**)**. There were also variations in terms of time spent in parks across the city. Among all the parks, the visitors to Al Rawdah Garden (35%) spent the most time there (1–2 h), followed by Al Nawras Garden (33%), Al Masarah Garden (31%), and Al Jamaa Garden (28%).

### Socio-demographic attributes and pattern of use and accessibility of urban gardens/parks

In this study, use and accessibility were largely influenced by socio-demographic attributes (gender, age, educational status, and occupation). Females (4.56) placed more importance on urban gardens/parks in comparison to males (4.26). Some females stated that they rarely visited urban gardens/parks alone; rather, they visited them with their partners, friends, or parents. On the contrary, males visited gardens/parks whenever they wanted. The use and accessibility of gardens/parks also varied in terms of age. Visitors aged from 20 to 30 and from 30 to 40 placed importance on the benefits obtained from gardens/parks. Most visitors aged from 20 to 40 were accompanied by partners, friends, or parents. The visitors within this age group brought their children with them so that the children could exercise in the open spaces and so that they could play with their children. The educational qualification of most visitors was a bachelor’s degree or higher (69% of the total park visitors). Most visitors with a bachelor’s or master’s degree visited parks to spend time with partners, friends, or relatives, to experience nature, and engage in physical activity. The use and accessibility pattern also varied by occupation. Visitors in business and government mostly used the urban parks. One government visitor (male, age 41) stated, “I visit this park (Al Jafali Garden) every weekend with my partner and two children. I don’t get time to spend time with my family outside, so we visit these parks to escape from the daily routine.” Table 5 represents the motivations of the park visitors to use urban parks in Jeddah city. As per results, it was found that the motivations behind the use of the urban parks were physical activity; mental refreshment; gathering people with family, friends, relatives, and children; and escape from daily life. The details of the motivations expressed by the park visitors are presented in Table 5.

**Table 5:** Motivations to visit urban parks in the Jeddah megacity

Benefits or purpose of park use	Visitor’s details			Name of the park
	Age	Gender	Perception of the visitors	
Physical Exercise	44	Male	“I have extreme blood sugar problems and I come to this park every day for physical exercise. I feel this park is very good for physical exercise.”	Al Jafali Garden
	26	Male	“I think this park is a good place for physical activities. I don’t think that one needs to go to a gym, as this park provides a beautiful environment for me. I have been coming here for the last four months.”	Al Masarah Garden
	31	Female	“I come to this park with my husband frequently to enjoy this place through physical exercise.”	Al Nawras Garden
Mental Refreshment	56	Male	“I frequently visit this park to spend time, as this is very close to my home.”	Al Jamaa Garden
	43	Male	“I come to this [park] every weekend to enjoy peace and patience. I belong outside the city, and this park gives me a lot of opportunities for mental refreshment from daily life.”	Al Rawdah Garden
Family Gathering	32	Female	“Every weekend I come to this park with my husband and two children. My children like this place very much; they enjoy this place.”	Al Rawdah Garden
	28	Male	“I don't get time to spend time with my family and whenever I get time, I come to here to spend time in this natural environment.”	Al Nawras Garden
	39	Male	“We have been coming to this park for the last 4 years. My wife likes this park and we spend a lot of time here.”	Al Jafali Garden
Escape from daily life	61	Female	“I got really bored at home, particularly during the pandemic. Now I feel better, as this park gives me lots of refreshment from my daily routine.”	Al Nawras Garden
	44	Male	“I have a deep attachment to this park, as my father used to come with me to this park. I think I have an attachment to many places in this park. I really feel better when coming to this park.”	Al Masarah Garden
Picnic	24	Male	“Whenever we get time, my friends and I come to this park. We bring food, spend time with each other, and have a picnic.”	Al Rawdah Garden
	26	Female	“My father and mother are both government employees; they don't get time to spend together. We try to come to this park once or twice a month; [we] arrange a family picnic and have lots of fun.”	Al Masarah Garden

### Statistical analysis

Table 6 shows the correlations among the benefits of urban parks. A natural environment had a positive correlation with other benefits. The natural environment largely influences other activities performed in urban parks. Particularly, a strong correlation was found between spending time with relatives (partners) and friends (0.933), reading (0.985), physical activity (0.905), experiencing solitude (0.814), and social relationships (0.647). The natural environment largely determined the other benefits obtained from the urban parks. Spending time with relatives (partners) and friends also showed a positive correlation with spending time with children, reading, physical activity, social relations, and sports.

**Table 6:** Correlations between activities and benefits obtained from gardens/parks

Activities/benefits	A	B	C	D	E	F	G	H	I	J	K	L
A	0.640	1										
B	0.289	0.575	1									
C	0.454	0.933^*^	0.467	1								
D	0.044	0.585	0.780	0.432	1							
E	0.284	0.336	0.810	0.885	0.770	1						
F	0.529	0.814	0.724	0.263	0.144	0.354	1					
G	0.737	0.985^**^	0.490	0.922^*^	0.443	0.195	0.346	1				
H	0.124	0.132	0.486	0.302	0.100	0.427	0.809	0.160	1			
I	0.510	0.905^*^	0.862	0.795	0.805	0.634	0.509	0.842	0.261	1		
J	0.397	0.136	0.668	0.692	0.473	0.926^*^	0.422	0.027	0.662	0.714	1	
K	00.552	0.647	0.909^*^	0.713	0.775	0.541	0.648	0.588	0.803	0.871	0.301	1
L	0.640	1										

*Correlation is significant at the 0.05 level (2-tailed)

**Correlation is significant at the 0.01 level (2-tailed)

Legend: A, walking; B, a natural environment; C, experiencing nature and its aesthetic beauty; D, spending time with relatives (partner) and friends; E, mental refreshment and relaxation; F, spending time with children (playing or traveling); G, experiencing solitude; H, reading; I, picnic; J, physical activity; K, sports; L, social relations.

Factor analysis was also carried out to examine the most significant benefits provided by the urban parks. Results are presented in Table 7. Five benefits (a natural environment, spending time with relatives (partners) and friends, picnics, sports, and social relations) played a significant role in explaining Factor 1. Spending time with relatives (partner) and friends, a natural environment, and social relations were given a high value. Factor 1 explained 53.35% of the total variance. From Factor 1, it can be stated that physical environment of the urban parks largely influences pattern of urban parks use in Jeddah city. In the case of Factor 2, three benefits were identified as crucial for visiting urban parks in the Jeddah megacity. These benefits were walking, experiencing nature and its aesthetic beauty, and experiencing solitude. Many visitors visit parks to escape from their daily routine, and these urban parks are largely used for physical fitness or to experience nature on the weekend and on holidays. Factor 2 explained 30.30% of the total variance. The factors affecting use of urban parks can be categorized under the cultural ecosystem services that promote well-being of the urban residents. Factor 3 identified four benefits (mental refreshment and relaxation, spending time with children (playing or traveling), reading, and physical activity) and explained 16.34% of the total variance (Table 7). Factor 3 can be categorized under mental and physical well-being of the people.

**Table 7:** Factor analysis for the main attributes behind visiting urban parks

Attributes	Factors		
	Factor 1	Factor 2	Factor 3
A	− 0.129	0.974	0.184
B	0.855	− 0.423	− 0.300
C	− 0.129	0.974	0.184
D	− 0.855	0.423	0.300
E	0.140	0.444	0.885
F	0.438	− 0.531	− 0.726
G	− 0.129	0.974	0.184
H	− 0.490	− 0.224	0.842
I	0.956	0.171	− 0.237
J	− 0.140	− 0.444	− 0.885
K	0.862	0.018	0.507
L	− 0.966	0.210	− 0.149
Eigen values	6.40	3.630	1.960
% of variance	53.34	30.30	16.340
Cumulative of %	53.34	83.65	100.000

Legend: A, walking; B, a natural environment; C, experiencing nature and its aesthetic beauty; D, spending time with relatives (partner) and friends; E, mental refreshment and relaxation; F, spending time with children (playing or traveling); G, experiencing solitude; H, reading; I, picnic; J, physical activity; K, sports; L, social relations

The Kruskal–Wallis test (K-S test) showed no significant difference in terms of valuations of perceived benefits from urban parks (the *P* value was < 0.05).

**Fig. 8:**
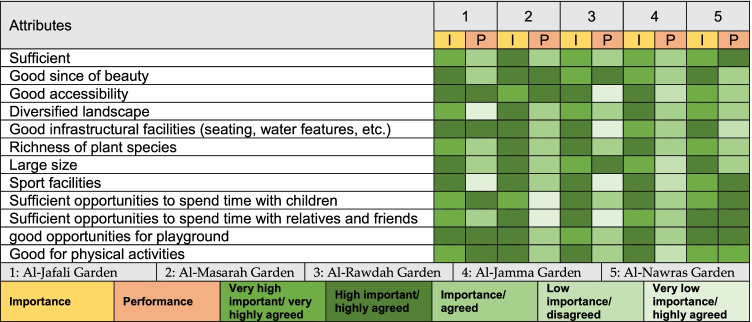
Comparison between perceived importance and performance

**Fig. 9:**
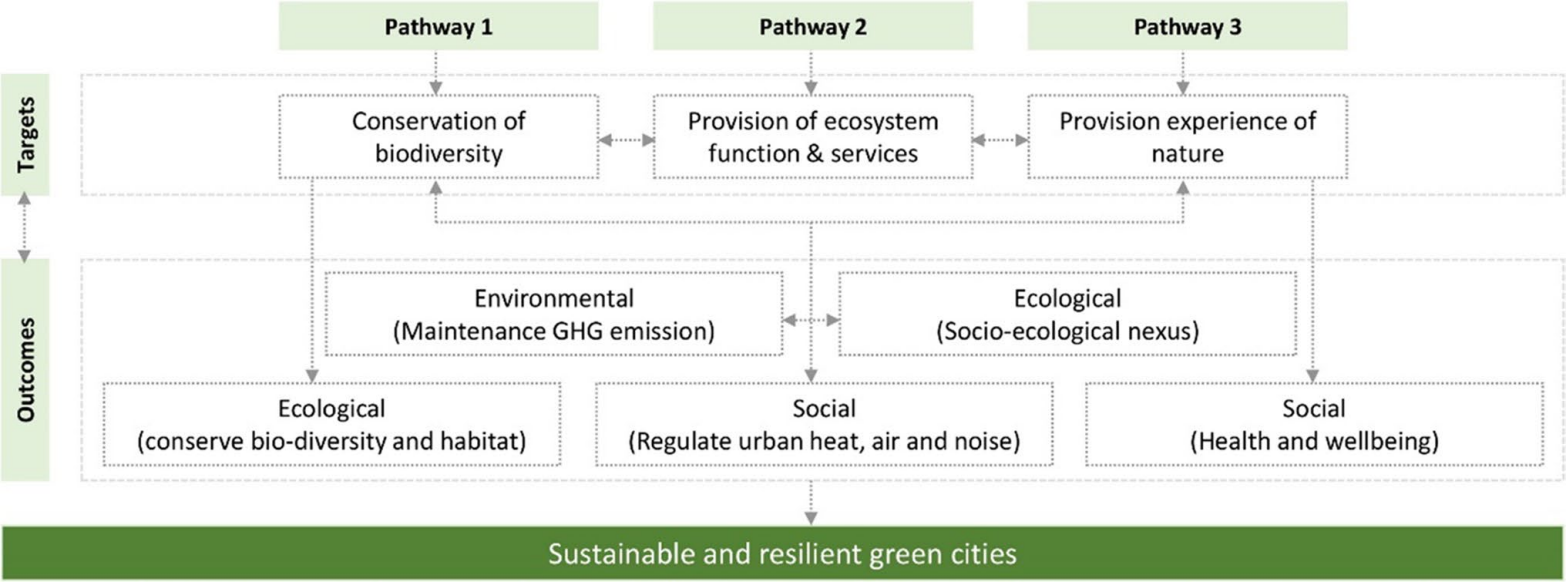
Nature-based solutions through greening cities and the achievement of sustainability

Figure 8 shows the importance and performance of attributes related to management strategies. Substantial discrepancies between importance and performance were reported in relation to certain attributes related to the management strategies of urban parks. As per the perception of the visitors, the visitors were not satisfied with the size of the urban parks, the sports facilities, or the playgrounds. The value of the BDI ranged from 0.54 to 0.88, with the highest BDI reported in Al Jamaa Garden (0.88), followed by Al Jafali Garden (0.83), Al Nawras Garden (0.75), Al Rawdah Garden (0.73), and Al Masarah Garden (0.54). All BDI values were positive, which clearly indicates that the benefits of the urban parks were perceived as important in all urban parks. Particularly, significant importance was attributed to spending time with relatives (partner) and friends, a natural environment, experiencing nature and its beauty, and physical activates (Fig. 9).

## Discussion

In this study, we examined the patterns of use and accessibility of urban gardens/parks in Jeddah. Results show that urban gardens/parks were mostly used for spending time with relatives and friends, followed by mental refreshment and relaxation, physical activity, spending time with children, and experiencing the natural beauty of the environment. Similar findings were also reported from other previous studies (Scopelliti et al. 2016; Liu et al. 2017a, b). As with other countries (such as Western countries), the urban parks in Jeddah are used for similar purposes, but there are differences in terms of cultural beliefs, religious difference, and weather. For example, Jeddah is characterized by a hot and humid climate where temperature reaches to 38 °C to 40 °C during summer. According to previous research studies, urban gardens and parks are mainly used for physical activity, social cohesion, and mental refreshment (Peters et al. 2010; McCormack et al. 2010; Khosravaninezhad et al. 2011). According to P.A.L and K.G.C.P (2015) and Sreetheran (2017), mental refreshment and relaxation were the most significant motivations for visiting urban parks, and the social aspects of urban parks have also been considered major reasons for visits (Moulay et. al. 2017). In urban areas, especially in large cities, contact with the natural environment and escape from one’s daily routine are significant benefits of urban parks (Razak et. al. 2016; Sirina et al. 2017), and this study showed that urban parks are considered crucial for experiencing nature and escaping from the daily hustle due to extreme temperatures, particularly during the summer season. In Jeddah, urban parks are not large areas, but they play a significant role in the well-being of urban citizens (e.g., providing physical and mental health benefits) due to otherwise insufficient opportunities for contact with nature. Thus, the urban gardens/parks in Jeddah are perceived as important for spending time in outdoor spaces (Addas and Maghrabi 2021b) and have been highly recognized for their benefits or cultural services, with our findings similar to those of previous research (Swamy and Devy 2010; Paul and Nagendra 2017; Swapan et al. 2017) (Table 8).

**Table 8:** Previous literature on the use, accessibility, and perceived importance of urban parks

Study area	Authors	Publication year	Major findings
Review paper	Kerishnan and Maruthaveeran	2021	The use of urban parks is largely influenced by the physical and social attributes of the people
Tokyo (Japan)	Guan et al	2021	The use of urban parks varies based on season
Copenhagen (Denmark)	Lindberg and Schipperijn	2015	Males reap the benefits of urban gardens/parks more than females
Taipei City (Taiwan)	Lin and Lin	2016	Parks had a significant impact on cooling and the urban microclimate, and they enhance the urban thermal environment
Rome (Italy)	Gratani et al	2016	Urban parks play a significant role in carbon sequestration
Kuala Lumpur (Malaysia)	Sreetheran	2017	Most of the respondents visited urban parks for fresh air and to relax and reduce stress
(Beijing) China	Amani-Beni et al	2018	Urban parks have a significant impact on the mitigation of the thermal environment and a cooling effect
Seoul	Jo et al	2019	Urban parks are considered significant determinants of carbon reductions
Los Angeles	Romolini et al	2019	There was a positive relationship between place attachment and the frequency of urban park visits
Shanghai (China)	Zhai et al	2018	A person visits urban parks mainly due to social and nature-oriented benefits. People visit parks often with their families and friends
Singapore	Zhang and Tan	2019	Attitudes and perceptions had a positive impact on the use of urban parks
Fuzhou (China)	Michelle et al	2021	Satisfaction with and perception of urban parks were largely influenced by the personality, health, and moods of the respondents
Leipzig (Germany)	Kabisch et al	2021	Visitors visit parks more frequently during summer heat waves
Taipei city (Taiwan)	Chang and Li	2014	Urban parks played a crucial role in maintaining the thermal condition of the city
Wuhan (China)	Liu et al	2017	The size and availability of services determine the visitors of urban parks
Shanghai (China)	Zhai et al	2018	Visits to urban parks were largely determined by social and nature-oriented motivations
Singapore	Zhang and Tan	2019	Attitudes, subjective perceptions, and accessibility affect the use of urban parks

Based on the interactions with the respondents, visitors older than 60 years placed a relatively high importance on, and spent more time in, parks for their contribution to physical and mental well-being. These findings are similar to those of previous studies performed by Mutiara and Isami (2012) in Jakarta, Schipperijn et al. (2010) in Denmark, and Paul and Nagendra (2017) in Delhi. The importance of urban parks was also higher for women who visited parks with their friends and partners. However, it has also been reported many times that women cannot visits parks due to their expected domestic duties (Basu and Nagendra 2021).

In Jeddah, urban parks/gardens are sources of multifunctional ecosystems and common resources in the city. These parks/gardens provide various recreational and provisioning services. Interactions between humans and parks/gardens represent a strong socio-ecological nexus that, on the basis of visitors’ perceptions and preferences, further assists the community-based management of these gardens/parks. Apart from recreational services, urban parks are also crucial in supporting livelihood strategies for marginalized communities (Basu and Nagendra 2021). Previous studies performed by Murwendo (2011) in Masvingo (Zimbabwe) and Stickler and Shackleton (2015) in Eastern Cape (South Africa) reported that urban parks provide diverse benefits that support citizens’ livelihoods. In other studies, urban parks were identified as areas that conserve bio-diversity by providing shelter and food to stakeholders (Mexia et al. 2018; Barth et al. 2015). Regarding this study, the livelihood strategies can be linked to marginalized communities to improve their lives.

The results show that city dwellers are highly dependent on gardens/parks. Accessibility to urban gardens/parks enhances not only urban environmental sustainability through temperature regulation but also the quality of life of urban citizens (Heidt and Neef 2008; Kothencz et al. 2017). Previous research has shown that the availability of per capita green spaces (PGGSs) in Jeddah is relatively low compared to other cities in Saudi Arabia such as Riyadh and Dammam (Addas and Maghrabi 2021a). Therefore, the per capita availability of green spaces must be enhanced at the neighborhood level to enhance the use and accessibility of urban parks. As per the results of this paper regarding importance and performance of urban parks, it was found that the performance of the attributes related to the management strategies was lower than the importance. It is thus clear that effective management strategies to meet the need of the urban residents require prioritization. The urban parks received relatively lower performance scores in areas such as accessibility, scenic beauty, size of the urban parks, and sports facilities. Therefore, it is urgent to focus on these management strategies to satisfy the needs of the urban residents. In previous studies, management strategies have been based on the performance of the river ecosystem services and public open spaces (Allan et al. 2013). In Jeddah, the local government must focus on the enhancement of facilities provided by the urban parks. The equipment and green coverage in urban parks must be enhanced as per respondents’ perceptions. Previous studies have also shown that successful management strategies gave priority to the satisfaction of the needs of the citizens (Hua and Chen 2019; Addas et al. 2021). In addition, accessibility to the urban parks must be enhanced to satisfy the needs of the park visitors (Wang et al. 2021).

### Policy implications for urban green space management

Previous studies have shown that the per capita availability of green space in the Jeddah megacity is much lower than in other cities such as Riyadh and Dammam (Addas and Maghrabi 2021a). For example, in Dammam and Riyadh, per capita green space is 5.4 and 1.18m^2^, respectively, whereas per capita green space was 0.5m^2^ in Jeddah. The per capita green space in Jeddah was thus ten times lower than Dammam and two times lower than Riyadh (Addas and Maghrabi 2021a). Our results show that, though the urban parks provided substantial benefits to visitors, there were also substantial discrepancies between the perceived importance and performance of their attributes. Therefore, the local authorities must focus on the creation of green spaces and the maintenance of existing green spaces to cope with climate change and to improve the well-being of urban citizens. In Saudi Arabia, the Ministry of Municipal and Rural Affairs and Housing (MoMRAH) is responsible for the maintenance of green spaces in cities. Apart from the MoMRAH, the maintenance of green spaces and the creation of new ones in cities can be promoted through the civic awareness of green spaces and of their contribution to human well-being, and through the active participation of local government. As per the guidelines of MoMRAH (1996), in Saudi Arabia, there must be one park for every 2500 to 5000 persons, and the per capita area allotted is 2–10 m^2^. The MoMRAH also states that public open spaces (POSs) must consider gardens and parks in their decision-making framework. It is important to note here that MoMRAH developed a master plan for Riyadh in 1970 to promote public green space in the city, and this master plan was able to achieve its goals. Such a master plan can be implemented for the Jeddah megacity, and it must be properly performed though effective measures. In Saudi Arabia, the Vision 2030 National Transformation Program (NTP) was implemented to increase per capita green spaces and to improve the quality of life of urban citizens in Saudi cities. Under the NTP, the Green Riyadh project was established to increase green cover across the city (planting 7.5 million trees). Such a green project can also be adopted for the Jeddah megacity to increase green coverage across the city at city and neighborhood levels.

### Limitations and future research directions

This study utilized a large scoping design to understand the patterns of use, accessibility, and attitudes in relation to urban park visitors. To the best of our knowledge, this is the first study in a Saudi context addressing the perceptions of urban parks from diversified perspectives. Despite this, two major limitations can be highlighted. Due to the COVID-19 pandemic, we could not conduct an extensive survey in the parks and a total of 409 visitors were surveyed from five parks. Thus, the sample size may not be representative of the entire city. A future study must consider an adequate sample size from the parks for an improved analysis. Second, from the field observations, only 12 activities were taken into consideration, but these parks are used for other activities as well. Therefore, a future study should consider these. Lastly, for selection of the urban parks, the effect of residential segregation was not taken into consideration. Future studies must thus take this into consideration. Regardless of these limitations, this study highlights the importance of urban parks in hot desert climates, such as that of Saudi Arabia.

## Conclusion

The present study presented an assessment of the use and accessibility of urban parks in Jeddah, Saudi Arabia, using a questionnaire survey and multivariate statistical analysis. Twelve activities or services were identified in five urban parks. The urban parks were mainly used for spending time with relatives (partners) and friends, followed by mental refreshment and relaxation, physical activity, spending time with children (playing and traveling), and experiencing nature and its aesthetic beauty. There were substantial seasonal variations in urban park use. The frequency and duration of park visits were higher and longer in the summer compared to the winter. During the summer season, most park visitors used the park once a week, whereas once-a-month visits were most common in the winter. Visitors spent more time in urban parks in the summer compared to the winter. The use of urban parks is determined by socio-demographic attributes such as age, gender, education, and occupation. Finally, from the results, it was reported that the performance of attributes related to the management strategies were relatively lower in importance. This shows that urban parks need to be improved to meet the needs of the urban residents. Therefore, it is essential to focus on the enhancement of urban parks management such as improvement of green coverage, equipment, and accessibility. The enhancement of management attributes perceived by the respondents not only can meet the needs of residents but will also be beneficial for climate mitigation strategies. Thus, planners and policy makers must focus on effective management as well as provision of services from the urban parks.

## Data Availability

The data that support the findings of this study are available from the corresponding author, Abdullah Addas, upon reasonable request.
